# Mechanism study of photo-induced gold nanoparticles formation by *Shewanella oneidensis* MR-1

**DOI:** 10.1038/s41598-019-44088-4

**Published:** 2019-05-20

**Authors:** Bo Chuan Huang, Ying-Chen Yi, Jo-Shu Chang, I-Son Ng

**Affiliations:** Department of Chemical Engineering, National Cheng Kung University, Tainan, 70101 ROC Taiwan

**Keywords:** Environmental biotechnology, Biotechnology

## Abstract

*Shewanella oneidensis* MR-1, a bioelectricity generating bacterium, is broadly used in bioremediation, microbial fuel cell and dissimilatory reduction and recovery of precious metals. Herein, we report for the first time that photo induction as a trigger to stimulate gold nanoparticles (Au@NPs) formation by MR-1, with wavelength and light intensity as two key variables. Results indicated that sigmoidal model is the best fit for Au@NPs formation at various wavelengths (with R^2^ > 0.97). Light intensity in terms of photosynthetic photon flux density (PPFD) critically influences the rate constant in the low-light intensity region (PPFD < 20), while wavelength controls the maximum rate constant in the high-light region (PPFD > 20). By deletion of Mtr pathway genes in MR-1, we proposed the mechanism for light induced Au@NP formation is the excitation effect of light on certain active groups and extracellular polymeric substances (EPS) on the cell surface. Also, the release of electrons from proteins and co-enzyme complexes enhance electron generation. To the best of our knowledge, this is the first-attempt to explore the effect of photo-induction on Au@NPs production by MR-1, which provides an alternative cost-effective and eco-friendly process in green chemical industry.

## Introduction

Gold nanoparticles (Au@NPs) are widely used in biomedicine and various biotechnological applications due to their unique magnetic and optoelectronic properties, quantum size effect and biocompatibility. This flexibility has high potential for applications as transistors, oscillators, in catalysis^1^, and has even received particular interest in biosensors, drug delivery, and as therapeutic agents^2^. Traditionally, metal nanoparticles were synthesized in a bottom up procedure from atoms and molecules to nanoscale particles, which still remains as the main strategy^3^. The first Au@NPs were produced by treating hydrogen tetrachloroaurate (HAuCl_4_) with citric acid in boiling water, where citric acid serves as both reductant and stabilizing agent^4^. By adjusting the ratio of gold-to-citrate, the particle size of Au@NPs could be controlled. Later, numerous chemical synthesis processes were derived from this method which is often dubbed as the art of gold nanoparticles synthesis^5^. However, the reaction inevitably uses toxic ingredients and solvents, which are not only harmful to humans, but also has severe negative impacts on the environment.

Bio-fabrication of nanoparticles provides an alternative eco-friendly process compared to traditional chemical synthesis. Biological synthesis of gold nanoparticles could be performed by bacteria, actinomycetes, fungi^6^, yeast^7^ and viruses^8^, which could reduce the Au^3+^ ions into Au° in nanoscale. Among the microorganisms, dissimilatory metal reducing bacteria of the genus *Shewanella* are capable of reducing Au^3+^ ions and generate Au@NPs^9,10^. *Shewanella* are gram-negative facultative anaerobic proteobacteria. It is acidophilic, and can survive under high concentrations of heavy metal ions aided by a specific mechanism called extracellular electron transport (EET). The mechanism of metal reduction has been well-studied in *Shewanella* and the c-type cytochromes (i.e., MtrA, MtrB, MtrC, CymA, and OmcA) play a critical role in metal reduction^11^. The complex structure forming Mtr (i.e., metal-reducing) pathway functions by promoting the electron transfer from inner cell membrane to the cell surface^12^. Besides, the electron transfer potential of MR-1 could be enhanced by the synthetic flavin pathway^13^. Its capability of bioelectricity generation and metal reduction showed great potential in microbial fuel cells (MFC) and bioremediation^14,15^. Thus, *Shewanella* species are prospective candidates for the biological reduction of metal ions.

*S*. *oneidensis* MR-1was first isolated from Oneida Lake, New York (Myers and Nealson 1988) and was reported to generate Au@NPs in the range of 2 to 50 nm^16^. In particular, the outer membrane proteins MtrC and OmcA were considered as the key proteins for metal reduction and in controlling of the size of nanoparticles^17^. On the other hand, MR-1 mutant strain without *mtr*C and *omc*A still formed Au@NPs, but the particle size decreased^18^. The other valuable metal nanoparticles including silver and copper nanoparticles could also be synthesized by *S*. *oneidensis*^19,20^. Besides MR-1, *S*. *xiamenensis* showed outstanding capability in azo-dye decolorization, and was reported to reduce metal ion to nanoparticles^21,22^.

Bio-fabrication of nanoparticles has beneficial characteristics like low toxicity, high purity, and biocompatibility. However, the rate of synthesis rate is relatively low and needs to be scaled up. To overcome this, culture condition optimization such as pH value, incubation time, temperature, and metal ion concentration for commercial applications has been studied^23^. Unfortunately, the yield of nanoparticle was still lower than that of chemical synthesis. Flavin-related compounds have been proved to enhance the production rate and yield of nanoparticles^24–26^, but flavin compounds are expensive thereby limiting large-scale production. Zhang *et al*., first described that light intensity could influence silver nanoparticle formation by biofilms of *S*. *oneidensis*^27^, but the effect of photo-induction including light intensity and wavelength in bio-fabrication of NPs has remained mysterious. To the best of our knowledge, the effect of light intensity on the biosynthesis of Au@NPs by *S*. *oneidensis* has not been investigated. In this study, the effect of light intensity and wavelength of light on Au@NPs synthesis by wild type and mutant strains (deletions of Mtr pathway genes like *mtr*A, *mtr*B, *mtr*C, and *cym*A) of *S*. *oneidensis* MR-1 has been investigated. Finally, the possible mechanism for photo-induction of Au@NPs by *Shewanella* has been explored.

## Results and Discussion

### The effect of light intensity on Au@NPs formation

**Figure 1:**
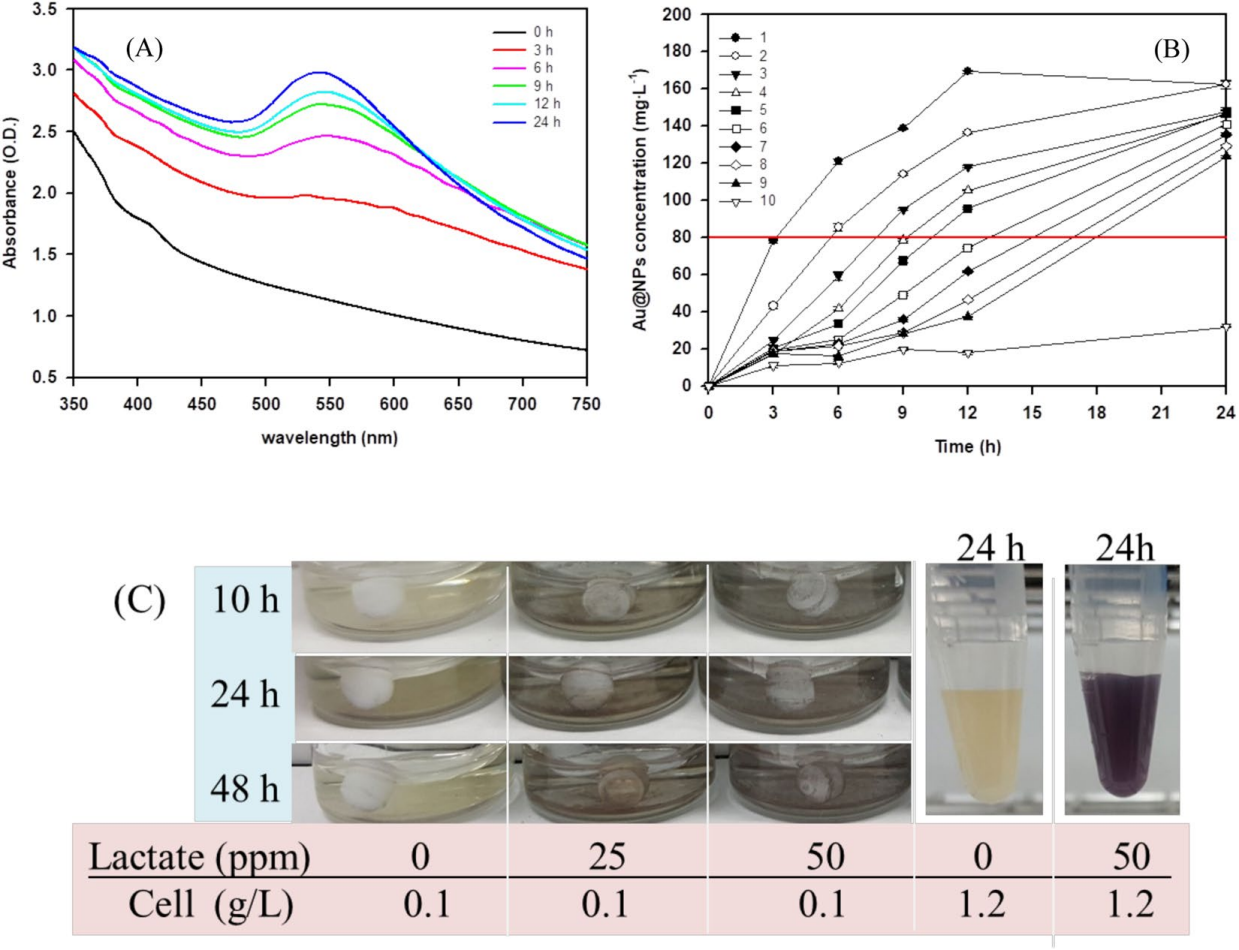
(**A**) The UV-Vis spectrum of the Au@NPs solution with time-lapse from 0 to 24 h showed the maximum peak is at 530 nm. The sample included 1.2 g/L biomass, sodium lactate 50 mM and Au^3+^ ion 300 mg/L with white light PPFD (113.9 μmol/m^2^/s). (**B**) The time-course of Au@NPs formation in different intensity of white light. The position number 1 to 9 refer to the ordering from the least to the highest distance apart from the light source where the light intensity (lux = lm/m2/s) at positions 1 to 9 is 11898, 7195, 4987, 3755, 3246, 2932, 2366, 1875, 1523, respectively; while 10 is dark control, and the horizontal red line standard for the concentration of Au@NPs at 80 mg/L. The correlation between distance and light intensity in different light source are shown in Table 1. All the experiments were performed in triplicate. (C) The lactate effect of Au@NPs formation under 0.1 g/L and 1.2 g/L cell, respectively.

Au@NPs formation by S. *oneidensis* MR-1 under standard white light illumination for 24 hours was verified by UV-Vis spectroscopy and the results are shown in Fig. 1A. Due to the surface plasmon resonance of Au@NPs, the gold nanoparticle (displayed in dark purple) has maximum absorbance at 530 nm. Within 24 hours, Au@NPs formation increased correspondingly with increase in time. The reduction of Au^3+^ to Au^0^ was investigated under illumination with different light intensities as achieved by varying the distance between the reaction tube and the light source (position 1 to 9 refer to the ordering from the least to the highest distance apart from the light source where the light intensity (*lux* = lm/m^2^/s) at positions 1 to 9 is 11898, 7195, 4987, 3755, 3246, 2932, 2366, 1875, 1523, respectively); while the light source at position 10 was considered as the dark reaction/ negative control. The effect of light intensity on Au@NPs formation is represented in Fig. 1B. As shown in Table 1, the light intensity in terms of lumen flux in unit area for white light was denoted by position 1 to 9, which is in the range between 11,898 to 1,523 lm/m^2^/s. Although the effect of light on Au@NPs formation by *Shewanella* has been reported^27,28^, detailed studies on the effect of wavelength and intensity has not been performed. Results indicated that light intensity was a critical factor for the formation of Au@NPs (Fig. 1B) and maximum Au@NPs production (i.e., 167 ppm at 12 h) was obtained with the highest light intensity (11,898 lm/m^2^/s) received at position 1. The light intensity at position 9 with 1,523 lm/m^2^/s produced the lowest amount of Au@NPs. The amount of Au@NPs generated was maximum with the irradiation time of 24 hr, which was consistent with the radiation effect on Au@NPs formation as reported previously^29^. By adjusting the light intensity, maximum Au@NPs could be produced by MR-1. We set the specific concentration of Au@NPs at 80 mg/L (shown in red line in Fig. 1B), for which position 1 at light intensity of 113.9 μmol/m^2^**/**s required approximate 3.2 h, position 2 at light intensity of 72.4 μmol/m^2^**/**s took 5.6 h, and finally the position 9 at light intensity of 16.8 μmol/m^2^**/**s needed 17.9 h (Table 2). It could be seen that photon accumulation (μmol/m^2^) is equal to the multiple of light intensity and reaction time, ranging from 1.18 × 10^6^ to 1.37 × 10^6^. Therefore, Au@NPs production by MR-1 was dominated by total photon accumulation under the given conditions. As lactate is the critical electron donor for Au@NP production by *Shewanella*^28^, we further analyzed the difference of Au@NPs with 0.1 g/L and 1.2 g/L biomass. As shown in Fig. 1C (Au@NPs formation represented by purple colors), the reducing capacity of lactate from 0 to 50 ppm with 0.1 g/L biomass is significantly lower than that with lactate at 50 ppm and 1.2 g/L biomass. Hence, the reduction of Au^3+^ to Au^0^ and further Au@NPs formation is mainly influenced by the biomass.

**Table 1:** The correlation between distance and light intensity with different light source.

Light source (Passed through color filter)		Standard white light (400–700 nm)		Red light (640–700 nm)		Blue light (425–490 nm)		Green light (500–560 nm)		Yellow light (570–620 nm)		Magenta light (400–500 nm and 660–700 nm)	
Position No.	Distance^a^	Light intensity^b^	PPFD^c^	Light intensity^b^	PPFD	Light intensity^b^	PPFD	Light intensity^b^	PPFD	Light intensity^b^	PPFD	Light intensity^b^	PPFD
1	12.0	11898	113.9	3592	35.5	6468	67	6702	67	7785	76.9	7298	74.3
2	16.0	7195	72.4	2349	18.6	4060	41	4262	42	5048	50.7	4754	47.8
3	20.0	4987	51.5	1650	16.3	2868	29	3035	30	3531	35.3	3409	33.5
4	24.0	3755	38.6	1222	12.1	2144	26	2272	24	2625	26.5	2581	25.4
5	26.0	3246	33.8	1075	10.7	1888	19	1982	20	2318	23.1	2263	22.3
6	28.0	2932	30.6	969	9.6	1673	17	1747	18	2076	20.6	2043	20.5
7	30.0	2366	24.5	779	7.8	1349	15	1371	14	1657	16.7	1654	16.1
8	38.0	1875	20.1	638	6.3	1120	11	1153	12	1354	13.6	1361	13.3
9	42.0	1523	16.8	537	5.3	923	9.5	927	10	1138	11.3	1129	11.2
10	Dark	ND	ND	ND	ND	ND	ND	ND	ND	ND	ND	ND	ND

^a^Distance is defined the straight distance from the light source by cm.

^b^Light intensity is defined as the lumen flux in unit area (*lux* = lm/m^2^/s)

^c^PPFD is defined as the photosynthetic photon flux in unit area (μmole/m^2^/s), in which wavelength of light range from 400 to 700 nm.

ND: not-determined.

**Table 2:** The light intensity, reaction time and accumulation of photon for Au@NPs formation of 80 mg/L at different distance from the light position.

Position	1	2	3	4	5	6	7	8	9
Distance (cm)	12	16	20	24	26	28	30	38	42
Light intensity (μmol/m^2^/s)	113.9	72.4	51.5	38.6	33.8	30.6	24.5	20.1	16.8
Reaction time (h)	3.2	5.6	7.7	9.2	10.3	13.0	15.0	17.0	17.9
Accumulation photon (μmol/m^2^ × 10^6^)	1.28	1.36	1.37	1.37	1.33	1.35	1.36	1.22	1.18

### Kinetic study and rate constant of Au@NPs formation

**Figure 2:**
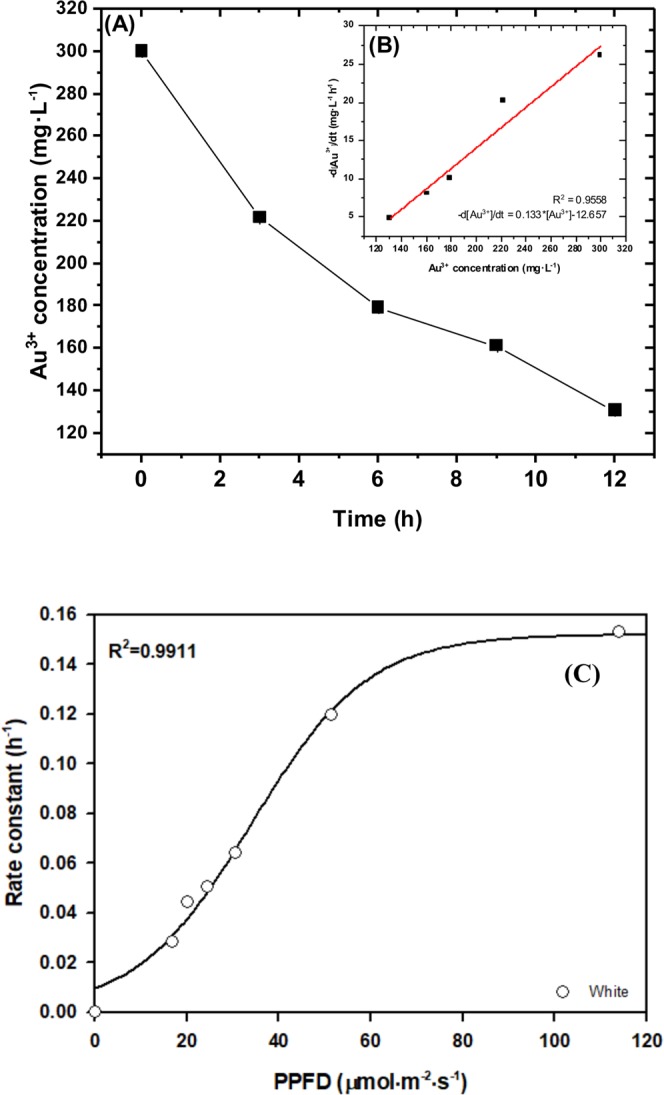
The kinetic analysis of Au^3+^ ion reduction by *S*. *oneidensis* MR-1. (**A**) Au^3+^concentration kinetic curve (**B**) the reaction rate versus Au^3+^ ion concentration. All experiments were conducted in triplicate at 25 °C, and the reaction mixture contains 1.2 g/L biomass, Au^3+^ ion 300 mg/L for initial concentration [Au^3+^]_0_ and lactate sodium 50 mM. In this figure, the sample was settled at position 1 (PPFD = 113.9 μmol/m^2^/s) (**C**) The correlation between white light intensity and rate constant. Light intensity is defined as photosynthetic photon flux density per unit area, called PPFD, with wavelength ranging from 400–700 nm. The calculation of rate constant is followed by differential equations (Eqs 2–4).

The relationship between the concentration of Au@NPs and absorbance followed the Lambert-Beer’s law^30^, providing quantitative analysis of nanoparticles in solution as:

1 $${\rm{Abs}}={\rm{\alpha }}\cdot [{\rm{Au}}@{\rm{NPs}}]$$

where Abs is the maximum absorbance of solution (in OD_530_), and α${\rm{\alpha }}$ is the product of extinction coefficient and length of light beam in the system, which served as a constant. [${\rm{Au}}@{\rm{NPs}}$] is concentration of gold nanoparticles in mg/L. Based on relative studies^31,32^, the biofabrication of metal nanoparticles under fixed light intensity can be described as a pseudo first order kinetic equation, represented by the following equations:

2 $${[{{\rm{Au}}}^{3+}]}_{{\rm{t}}}={[{{\rm{Au}}}^{3+}]}_{0}-{[{\rm{Au}}@{\rm{NPs}}]}_{{\rm{t}}}$$

3 $${[A{u}^{3+}]}_{t}={[A{u}^{3+}]}_{0}{e}^{-kt}$$

4 $${r}_{A{u}^{3+}}=-\frac{d{[A{u}^{3+}]}_{t}}{dt}=k{[A{u}^{3+}]}_{t}$$

where [Au^3+^]_0_, [Au^3+^]_t_ is the concentration of Au^3+^ion (mg/L) at initial stage and time *t*, respectively. [Au@NPs]_t_ represents the concentration (mg/L) at time *t* (h), and *k* is rate constant (h^−1^). Figure 2A shows that the consumption of Au^3+^ ion is in exponential decay trend which is consistent with Eq. 3. On the other hand, the relationship between reaction rate and Au^3+^ ion concentration can be properly fitted by linear regression with R^2^ = 0.9558 as shown in Fig. 2B, indicating that the overall reaction rate law follows Eq. 4 and the rate constant *k* is 0.133/h. From the above results, we confirmed that the reduction process obeyed pseudo first-order kinetics.

As light intensity strongly affected Au@NPs formation, a hypothesis of rate constant which obeyed a function of light intensity and wavelength was simulated in Fig. 2C. According to the experiment data in white light condition, sigmoidal model was the best fit for the data with high correlation (R^2^ = 0.9911) between rate constant and light intensity. The rate constant of light intensity combined with wavelength effect would be interpreted in the next section.

### The effect of light wavelength on Au@NPs formation

**Figure 3:**
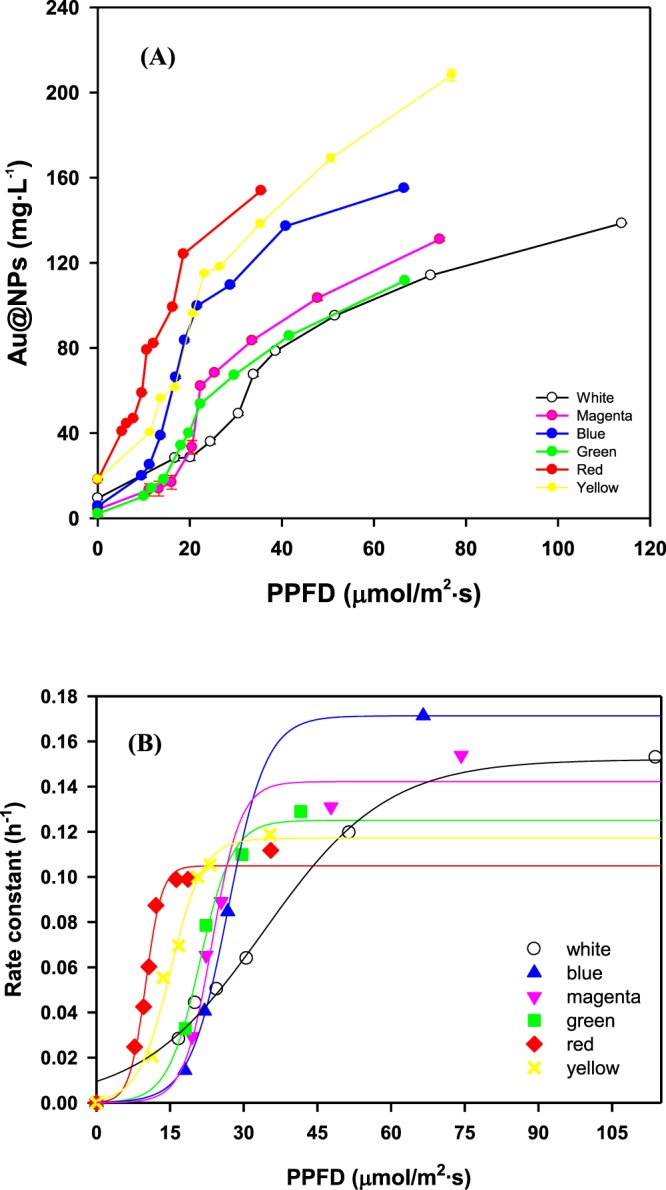
(**A**) Au@NPs formation by *S*. *oneidensis* MR-1with wavelength effect after 12 h. (**B**) Rate constant of Au@NPs formation by *S*. *oneidensis* MR-1 in various wavelength and PPFD within 12 h. The different wavelength of light exposure was obtained using colored filter to separate visible spectra from 400–700 nm for white light, 640 to 700 nm for red light, 425 to 490 nm for blue light, 500 to 560 nm for green light, 570 to 620 nm for yellow light, 400 to 500 and 660 to 700 nm mixture for magenta light. The light intensity was adjusted by distance from light source to reaction and the correlation between distance and light intensity in different light source were shown in Table 1. All the experiments were conducted in triplicate.

Besides light intensity, wavelength is another critical factor for Au@NPs formation. The light intensity in illuminance unit (*lux*) and photosynthetic photon flux density (PPFD, μmol/m^2^/s) for different wavelength are shown in Fig. S1. The rates of Au@NPs production at the initial stage with different light wavelength are as follows: red > yellow > blue > green > magenta > white light (Fig. 3A). A threshold value of 15 to 20 PPFD worked depending on the wavelength of light source. As shown in Fig. 3B, the fitting data from different wavelength by sigmoidal curve perfectly matched the behavior of rate constant depending on PPFD with rate constant *k* (h^−1^) as described in Eq. 5:

5 $$k=\frac{a}{1+{e}^{-(\frac{x-x0}{b})}}$$

where *a* is the maximum rate constant for *k*_*max*_ (h^−1^), *b* is a steep coefficient describing the sensitivity to the specific wavelength, and *X*_0_ is the requirement of photon flux density for half maximum rate constant. The maximum rate constant *a*, correlated to the wavelength in ascending order, consistent with the experimental results at high light intensity. It implied that wavelength determines the maximum rate constant rather than light intensity. As shown in Table 3, maximum rate constant for different wavelengths are as follows: blue at 0.171/h > white at 0.152/h > magenta at 0.142/h > green at 0.125/h > yellow at 0.117/h > red light at 0.105/h. Parameter *b*, in terms of accumulative photon per unit (μmol/m^2^**/**s, PPFD), controls the rate slope. With increase in *b*, the slope gets smoother. Therefore, the smooth change in rate constant occurred at white light while the steep change happened in red light. Moreover, *X*_0_ as a requirement of the photon flux density for half maximum rate constant, or presented as the transition point of sigmoidal curve, had a significant difference in broad wavelength (i.e., white light at 34.09 PPFD), followed by shorter wavelength (i.e., blue light at 21.58 PPFD) to longer wavelength (i.e., red light at 10.02 PPFD). As summary the effect of light wavelength in terms of photon flux density which is in the order of white light > blue light > green light > red light.

**Table 3:** Parameters involved in sigmoidal curve $k=\frac{a}{1+{e}^{-(\frac{x-x0}{b})}}$.

Light source	Wavelength (nm)	Maximum rate constant (*a*, h^−1^)	Steep coefficient (*b*, PPFD)	Photon flux density for half maximum rate constant (*X*_0_, PPFD)	R^2^
White	400–700	0.1520	12.555	34.0908	0.9910
Red	640–700	0.1049	1.6539	10.0168	0.9871
Yellow	570–620	0.1172	3.2892	15.0811	0.9852
Green	500–600	0.1250	3.3125	21.0752	0.9922
Magenta	400–500 660–700	0.1422	2.9037	23.5660	0.9764
Blue	425–490	0.1713	2.1442	21.5807	0.9990

**Figure 4:**
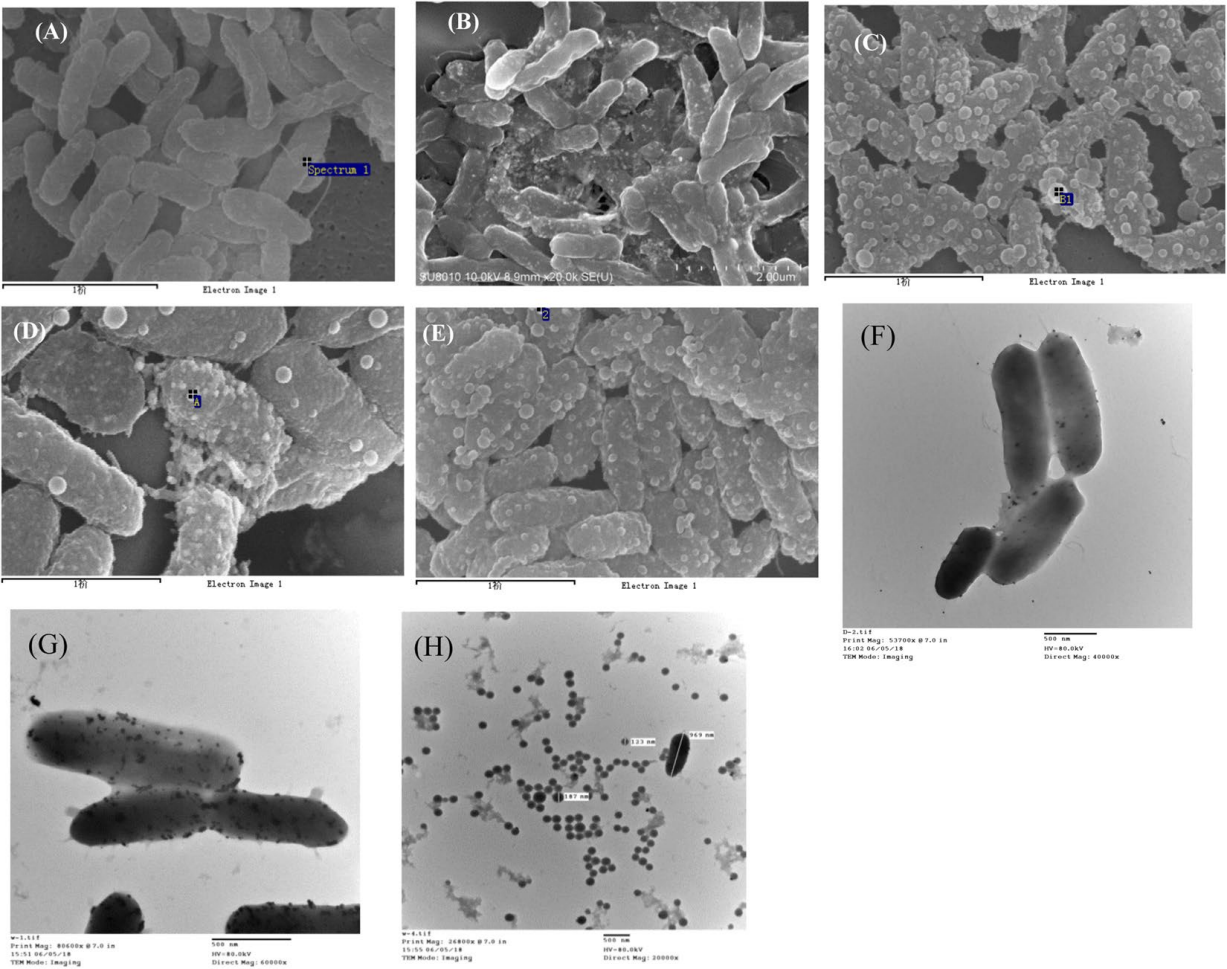
SEM and TEM images of nanoparticle formation by *S*. *oneidensis* with different wavelength of light exposure at 25 °C for 12 h. SEM: (**A**) dark (**B**) white light (**C**) red light (**D**) blue light (**E**) green light. The black line below the image is the scale for 1μm. TEM: (**F**) dark (**G**) white light and (**H**) Au@NPs extracted from cell.

We further confirmed the Au@NPs formation on cell surface via SEM and TEM analysis. From SEM results shown in Fig. 4A–E, Au@NPs production in dark was much lower than that of photo-induced experiments. Besides, more Au@NPs could be observed on the cell surface under red, blue and green as shown in Fig. 4C–E, which is consistent with EDS scanning results shown in Fig. S2. Moreover, the significant difference in Au@NPs formation under dark conditions (Fig. 4F) and white light induction (Fig. 4G,H) are shown in TEM images. The previous results shown in Fig. 1A represented the tailing at high wavelength and anisotropic growth of Au@NPs on the cell which is consistent with *Shewanella haliotis*^33^. However, the Au@NPs were of uniform size and spherical shape when collected by ultrasonication, and the normal distribution of Au@NPs presented in Fig. 4H was 15 nm, that is similar to the result by other *Shewanella* under pH control^34^.

As shown in Fig. 4C–E, the formation of membrane vesicles (MVs) is a stress response from *Shewanella* and it is composed of outer-membrane or periplasmic proteins in order to interact effectively with the substrate in the environment. Absorbance of Au@NPs and SEM images indicates that the highest Au@NPs are formed under red light which also shows the most MVs; however, there are no Au@NPs and MVs in dark conditions. We assumed that light would trigger the functional groups present on the outer membrane and stress the proteins resulting in MVs. So, the higher of MVs, the more Au@NPs produced.

### Effect of the deletion of Mtr pathway genes

**Figure 5:**
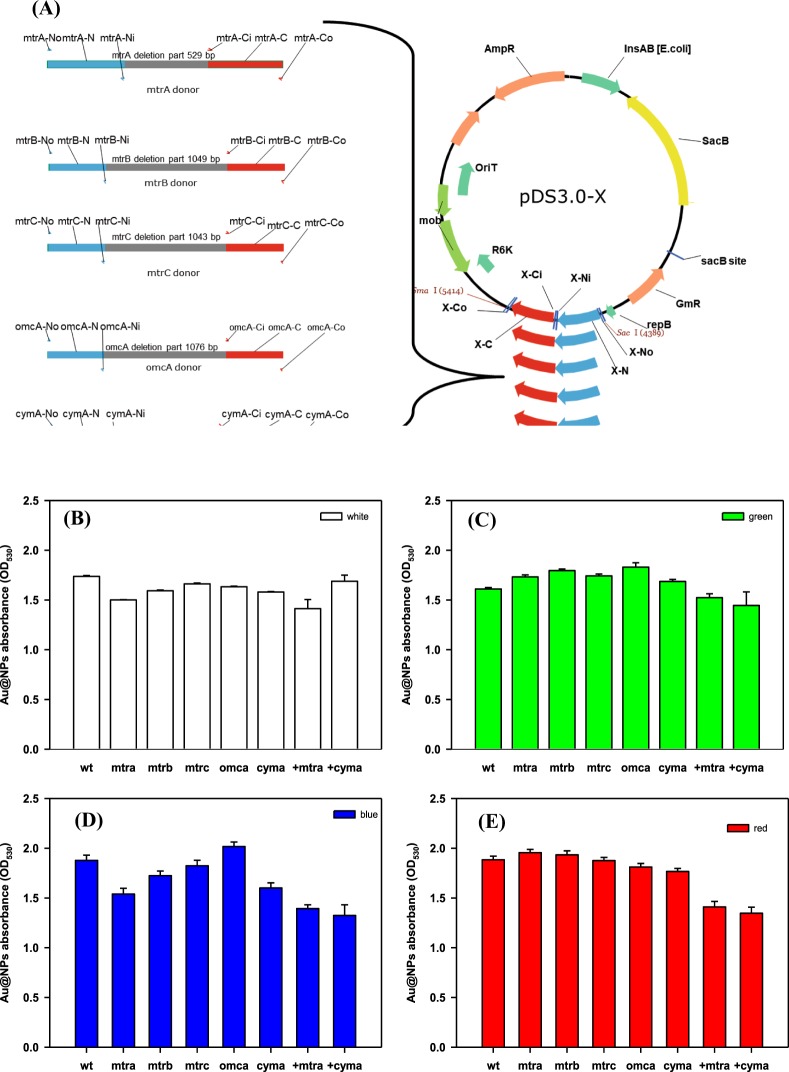
(**A**) Graphical scheme of gene knockout in *S*. *oneidensis* MR-1. Au@NPs formation by *S*.*oneidensis* mutant strains under exposure of different wavelength of light (**B**) white, (**C**) green, (**D**) blue and (**E**) red for 9 hr. WT: wild type strain, the other terms are the knockout gene strains, and the last two items are the complement of *mtr*A and *cym*A gene in the wild type strain. All the samples contained broth (OD_600_ = 1), Au^3+^ ion 300 mg/L and lactate sodium 50 mM for initial concentration, and followed by exposure to the specific wavelength light at the same intensity (PPFD = 50 μmol/m^2^/s).

The graphical scheme of gene knockout in MR-1 is shown in Fig. 5A. Deletion of the denoted genes slightly decreased Au@NPs formation in white light (Fig. 5B). Comparing the deletion mutant and supplementary effect of *cym*A and *mtr*A in the corresponding deletion mutants, it is noted that CymA, an inner membrane cytochrome, plays a crucial role in Au@NPs formation as well as MtrA. Previous studies mentioned that the outer membrane cytochrome MtrC play a crucial role in Au(III) reduction and nanoparticle nucleation^18,24^. However, we found the Au@NPs formation was highly influenced by different wavelengths of light in terms of green light (Fig. 5C), blue light (Fig. 5D) and red light (Fig. 5E) than in the various knockout strains (i.e., Δ*mtrA*, Δ*mtrB*, Δ*mtrC*, Δ*omcA* and Δ*cymA*). Except for Δ*omc*A strain, other knockout strains showed significantly decreased Au reduction capability under blue light. In contrast to our expectation, supplementation of *mtr*A and *cym*A genes to the corresponding mutants did not rescue the Au reduction ability but even reduced Au@NPs formation in green, blue and red light. The deletion of a single Mtr protein or altering its expression may not significantly decrease Au@NPs formation. The Mtr pathway, including MtrA, MtrB, MtrC, CymA and OmcA play important roles in metal reduction, and has been well-studied. However, the gene paralogs of MtrC and MtrA (MtrF and to a smaller extent OmcA) are able to form functional Mtr complexes, and can functionally replace MtrC^35,36^. When *mtr*C has been knocked-out, paralogs of *mtr*C gene expression could complement the deleted Mtr protein maintaining metal reduction. Therefore, MtrC deletion alone would not affect NPs production.

**Figure 6:**
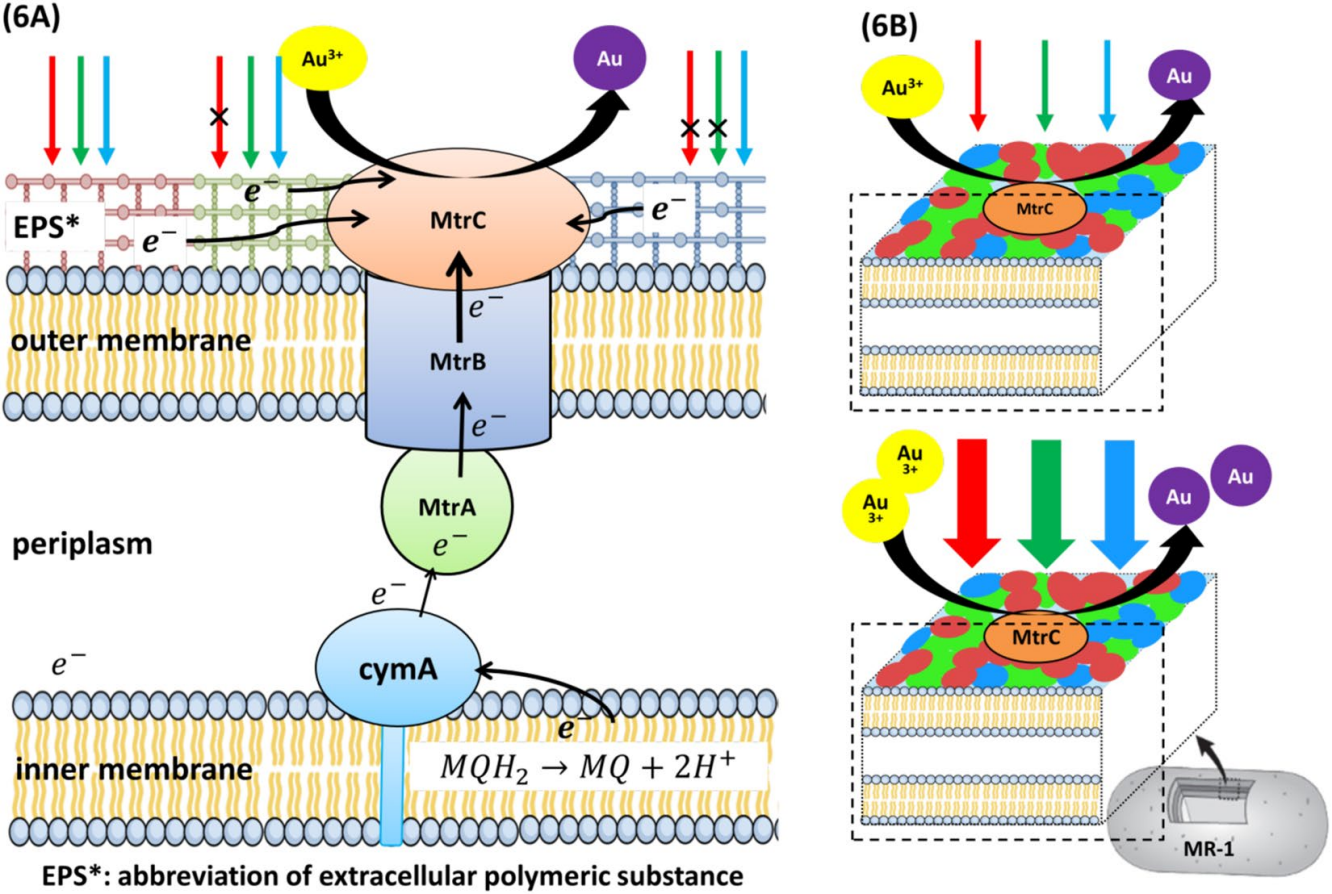
(**A**) The scheme for the mechanism of gold nanoparticle fabrication by photo-induction in *S*. *oneidensis* MR-1. (**B**) The correlation between light intensity and the quantity of bio-fabricated Au@NPs. Description: red, green and blue patches on the outer membrane refer to extracellular polymeric substances (EPS). Arrows with red, blue and green colors serve as the light source with different wavelength. Thickness of arrow in (**B**) indicates the amount of light intensity (photosynthetic photon flux density, PPFD).

According to the aforementioned results, we assumed that the cell surface of MR-1 might contain specific groups/active groups or substances that are activated by specific wavelengths of light. After activation, the proteins may catalyze electron generation or release electron by themselves and initiate the electron transfer chain. Figure 6A shows the hypothesis supporting the effect of photo-induction on gold nanoparticle fabrication in *S*. *oneidensis* MR-1. The red, green and blue patches on the outer membrane refer to extracellular polymeric substances (EPS) that can absorb light at different wavelengths and transport electron to the outer membrane cytochromes. When the light wavelength is shorter, its energy is higher that can expand the range of surface groups for photo-induction. Light intensity (photosynthetic photon flux density, PPFD) is depicted as the thickness of the arrow in Fig. 6B. Increase in photon density would possibly increase the number of activated surface groups to promote electron transfer; therefore the quantity of activated surface groups is proportional to the quantity of photons or light intensity. Different surface groups have their specific energy gap to overcome the activated energy that is similar to the “photoelectron effect”. In this case, when *S*. *oneidensis* is exposed to a high energy light beam, the external energy could help overcome the energy gap of most surface groups, leading to the improvement in Au@NP generation and the rate constant in high light intensity region (PPFD > 20). This phenomenon has also been applied in the microbial photo-electrochemical system (MPC) by *Shewanella* and hematite nanowire photoanode^37^. However, the total number of the so called surface groups is limited, and even if light intensity increases, the total sum of activated surface groups will reach a maximum as determined by the wavelength. As a result, the total sum of activated surface groups and the maximum rate constant under blue light are greater than that in red light. In the low-light region (PPFD < 20), the reasonable explanation is that this phenomenon is attributed to the nature of the surface group itself. As electron transfer in bioelectricity generating microbes would be affected by cell-surface exposed conductive proteins, some groups are sensitive to low light quantum density, and some are not^38^.

Previous research showed that extracellular polymeric substance (EPS), including protein and carbohydrates were involved in metal nanoparticle formation^39,40^. Therefore, we assumed that EPS had some kind of light sensitive protein abundantly dispersed on the cell surface, for example, cytochrome. Gene knock-out experiments indicated that the main cytochrome in Mtr pathway plays a role in the gold nanoparticle formation and reduction of unyielding chemicals like dinitrotoluene^41^ that is consistent with *S*. *oneidensis* MR-1 which is known for metal reduction with abundant c-type cytochromes. On the other hand, silver nanoparticles produced by MR-1 have been demonstrated with relation to the genes of Mtr pathway^17,42^. However, the deletion of these genes did not cause any significant effect on Au@NPs production by MR-1. This could be due to the diversity of cytochromes in *S*. *oneidensis*. Addition or removal of one kind of cytochrome does not effectively interfere with the light effect on Au@NPs formation. On the other hand, previous studies also reported that the electron shuttle, flavin and its derivative, such as FAD and FMN, can also be excited by the UV and blue light (430~450 nm) to improve the oxidation performance, but it only explains the phenomenon in blue light region. Flavin synthesis by wild-type *S*. *oneidensis* MR-1 was shown to be 1.31 ± 0.2 μM after incubation in LB medium for 16 h^43^, which was assumed to produce approximately 0.17 mg/L of Au@NPs. This is drastically lower than that of high light stress where 180 mg/L of Au@NPs was produced by MR-1 at 12 h. Though flavin redox could affect Au@NPs formation, the influence was critically lower than a physical stress like light effect. Therefore, flavin effect is considered negligible. To sum up, we consider that the active surface groups might also involve extracellular polymeric substances (EPS), including specific proteins and flavin derivatives occurring as co-enzymes. The details of EPS and related proteins (or enzymes) affecting Au@NPs formation still needs further investigation.

## Conclusions

We report for the first time the mechanism for Au@NPs formation via accumulation of photons (PPFD) based on light intensity and wavelength. The rate constant of Au@NPs formation is dominated by light intensity at low PPFD, while the wavelength controls the final rate constant in the high light region. By applying sigmoidal model, the rate constant, steep coefficient and affinity of photon flux density were predicted for Au@NPs formation under different light intensity and wavelength. MR-1 strains defective in one of the Mtr pathway genes showed only a slight decrease in Au@NPs formation depending on the wavelength.

## Materials and Methods

### Materials

The chloride hydrate salt of Au (HAuCl_4_·3H_2_O) was used to prepare Au^3+^ ion solution, which was purchased from Alfa Aesar (Aldrich 254169) and dissolved in distilled water at final concentration of 1000 ppm. Sodium lactate was purchased from Showa (G1510E). For scanning electron microscopy, formvar solution was purchased from Sigma (09823), and tert-butanol was purchased from Shimakyu Chemical Co. Ltd.

### Bacterial culture

*S*. *oneidensis* MR-1 was used for the production of AuNPs in this study. The bacteria was grown in Luria–Bertani broth containing yeast extract (5 g/L), sodium chloride (10 g/L), and tryptone (10 g/L). Both the native and recombinant strains were maintained at 4 °C on LB plate, and a single colony was inoculated into 2 mL of LB medium and cultured at 30 °C and 150 rpm for 12 h. For AuNPs production, 1% (*v/v*) of pre-culture was transferred into a 50-mL flask containing 10 mL LB medium and grown at same conditions.

### Construction of marker-free deletion mutants

Marker-free mutations in *S*. *oneidensis* were generated by in-frame deletion with modification^31^. Mtr-pathway related genes such as *mtr*A (SO_1777), mtrB (SO_1776), *mtr*C (SO_1778), *cym*A (SO_4591), and *omc*A (SO_1779) were chosen. Fragments with a length of 500 bp for upstream and downstream elements were amplified by PCR with designed primers as listed in Table S1. The suicide plasmid pDS3.0 with the *Sac*B gene (levansucrase) was digested with *Sma*I and *Sac*I, ligated to PCR products, and was transformed into *E*. *coli* WM3064 via heat shock. The harvested MR-1 was mixed with WM3064 with plasmids for conjugation. The cell mixture was plated on LB containing 10% (*w/v*) sucrose for activation of the suicidal gene and all gene deletions were verified by colony PCR.

### Biofabrication of Au@NPs under photo-induction

Cells were harvested by centrifugation at 7,000 × *g* for 10 min and washed twice with deionized water to obtain a final biomass concentration of 1.2 g/L. The biomass was resuspended in sodium lactate and Au^3+^ solution at a final concentration of 50 mM and 300 mg/L, respectively. The mixture was incubated in a closed vial with 80 rpm stirring at room temperature (approximately 25 °C) with a light intensity ranging from 0 to 120 μmol/m^2^/s, as determined by a photometer (Licon, Li-250A, Taiwan) for 24 h. Sample tubes covered by two layers of aluminum foil served as dark control in the experiment. Different wavelengths of light exposure was used; colored filter was used to separate visible spectra into light with wavelength range of 400–700 nm for white light, 640 to 700 nm for red light, 425 to 490 nm for blue light, 500 to 560 nm for green light, 570 to 620 nm for yellow light, 400 to 500 and 600 to 700 nm mixture for magenta light. Light intensity was adjusted by tuning the distance between the light source and reaction tube and the correlation between distance and light intensity in different light sources are shown in Table 1. For light intensity measurement in color light, the same photometer (as previous mentioned) was covered with the colored filter and fixed at the specific position to vary the light intensity as specified by the distance from the white light source. For evaluating the reducing capacity of lactate, 0, 25 and 50 ppm of sodium lactate was added to test the Au@NPs formation by 0.1 g/L and 1.2 g/L cell, respectively.

### Measurement of Au@NPs by UV–VIS spectroscopy

A 0.2 mL reaction mixture was taken from each sample at 0, 3, 6, 9, 12, and 24 h, and was further scanned by UV-VIS spectroscopy (Molecular Devices, SpectraMax, USA) at wavelength from 350 to 750 nm. The color change from pale yellow to purple indicates the formation of Au@NPs. The absorbance at 530 nm is due to surface plasmon resonance of Au@NPs^44^. The standard curve of Au@NPs was prepared by reacting 100 ppm HAuCl4 with 3.5 mM citrate sodium at 100°C for 15 mins. The calibration curve between OD530 and Au@NPs (mg/L) were converted by the equation Y (OD_530_) = 0.0111 × (mg/L) + 0.0674.

### Scanning electron microscopy (SEM)

The samples were fixed in 2.5% (*w/v*) glutaraldehyde for 2 h and washed three times with phosphate buffer (0.1 M, pH 7.4). The sample (100 μL) was carefully dropped onto a silicon wafer for 1 h and washed with phosphate buffer for 3 times. The cells were dehydrated in a series of ethanol washes with increasing ethanol concentration from 30 to 100%. The samples finally were immersed in tert-butanol and dried by lyophilization (Kingmech, Taiwan) for 2.5 h. Dehydrated samples were analyzed using SEM (Hitachi SU8010, Japan).The energy diverse spectrometer (EDS) coupled with SEM was used to determine the valence state of gold.

### Transmission electron microscopy (TEM)

The sample was centrifuged and wash with 0.1 M phosphate buffer for 2–3 times. The pellet was re-suspended in 0.5 mL of 2.5% glutaraldehyde and was frizzed at 4°C for half an hour. The suspension was dehydrated with a series of ethanol washes with increasing ethanol concentration, and was dispersed well in 4 mL water-free ethanol by ultrasonication for 30 min. A drop of solution was placed on a carbon-coated copper grip which was then dried at room temperature, while the residual solution was removed with paper. The TEM (Hitachi H-7500, Japan) of the sample gives information on the morphology, size and shapes of Au nanoparticles.

### Ethics approval and consent to participate

All the authors have read and agreed the ethics for publishing the manuscript.

## Supplementary information

Supporting Figure and Table
