# Supplementary material for: Mechanism study of photo-induced gold nanoparticles formation by Shewanella oneidensis MR-1
Source: Sci Rep. 2019 May 20;9:7589. doi: 10.1038/s41598-019-44088-4 (PMC6527576; doi:10.1038/s41598-019-44088-4)
Supplement: Supplementary file 1 — Supporting Figure and Table [file 41598_2019_44088_MOESM1_ESM.docx]

Supporting information

Mechanism study of photo-induced gold nanoparticles formation by *Shewanella oneidensis* MR-1

Bo Chuan Huang, Ying-Chen Yi, Jo-Shu Chang, I-Son Ng^*^

Department of Chemical Engineering, National Cheng Kung University,

Tainan 70101, Taiwan, ROC

*Corresponding author: I-Son Ng

E-mail: [yswu@mail.ncku.edu.tw](mailto:yswu@mail.ncku.edu.tw) ; ORCID: 0000-0003-1659-5814

Co-authors:

Bo Chuan Huang (email: [wl010706@gmail.com](mailto:wl010706@gmail.com) )

Ying-Chen Yi (email: [chan50515@gmail.com](mailto:chan50515@gmail.com) )

Jo-Shu Chang (email: [changjs@mail.ncku.edu.tw](mailto:changjs@mail.ncku.edu.tw); ORCID: 0000-0003-3469-9553)

Tel: +886-62757575-62648; Fax: +886-62344496;

Table S1 Bacterial strain, plasmid and primer list

| **Material** | **Genotype or description** | **Sources** |
| --- | --- | --- |
| **Strains** |  | |
| *Shewanella oneidensis* MR-1 | *S. oneidensis* MR-1 wild type | Lab stock |
| *E. coli* DH5α | 4,507,030 bp, F^–^*endA1* *glnV44* *thi-1* *recA1* *relA1* *gyrA96* *deoR* *nupG* *purB20* φ80d*lacZ*ΔM15 Δ(*lacZYA-argF*)U169, hsdR17(*r_K_*^–^*m_K_*^+^), λ^–^ | Lab stock |
| *E. coli* WM3064 | thrB1004 pro thi *rps*L *hsd*S lacZΔM15 RP4-1360Δ(araBAD)567ΔdapA1341::[ermpir] | Provided by Hai-Chun Gao |
| **Plasmids** |  | |
| pDS3.0 | Amp^R^ , Gm^R^ , derivative from suicide vector pCVD442 | Lab stock |
| pDS3.0-sfGFP | pDS3.0 containing fragment of HR and sfGFP | Lab stock |
| pDS3.0-*mtr*A | pDS3.0 containing fragment of *mtr*A-N and *mtr*A-C | This study |
| pDS3.0-*mtr*B | pDS3.0 containing fragment of *mtr*B-N and *mtr*B-C | This study |
| pDS3.0-*mtr*C | pDS3.0 containing fragment of *mtr*C-N and *mtr*C-C | This study |
| pDS3.0-*omc*A | pDS3.0 containing fragment of *omc*A-N and *omc*A-C | This study |
| pDS3.0- *cym*A | pDS3.0 containing fragment of *cym*A -N and *cym*A -C | This study |

| **Primers** | Sequences (5'→3') | purpose |
| --- | --- | --- |
| mtrA-No | AAGAGCTCCCAGTTATCCGGTAACGTGAGGT | mtrA-N HR |
| mtrA-Ni | AAGCGTATGTCTTGGAATGGCGGTCACAGCTATTACAGCGCTAAGGAGACGAGA | mtrA-N HR |
| mtrA-Co | AACCCGGGGTTGGGACAAATTGGGAAGCCTA | mtrA-C HR |
| mtrA-Ci | TCTCGTCTCCTTAGCGCTGTAATAGCTGTGACCGCCATTCCAAGACATACGCTT | mtrA-C HR |
| mtrB-No | AACCCGGGACCAAGTACACGTCGCAAAAGATCC | mtrB-N HR |
| mtrB-Ni | GTTAAGCGAAGACTCGTCTCCTTAGCGCTGTAAT | mtrB-N HR |
| mtrB-Co | AAGAGCTCTATCGGTATAATCATCTAACGCG | mtrB-C HR |
| mtrB-Ci | ATTACAGCGCTAAGGAGACGAGTCTTCGCTTAACAGGTAGTTA | mtrB-C HR |
| mtrB-F | GATCACTCTAGCGTTATTAGCCA | colony PCR |
| mtrB-R | GGCATTGGCAGAGCGAATATC | colony PCR |
| mtrC-No | AAGAGCTCCCCCTGCTTGGGCAAATTACA | mtrC-N HR |
| mtrC-Ni | TTAGCAGACGGAACGACCGTTCCCGTTTTCACAAAGGCAAACAACATGCAGGCT | mtrC-N HR |
| mtrC-Co | AACCCGGGGCAATGCCAAACCTATGCAGGGA | mtrC-C HR |
| mtrC-Ci | AGCCTGCATGTTGTTTGCCTTTGTGAAAACGGGAACGGTCGTTCCGTCTGCTAA | mtrC-C HR |
| omcA-No | AAGAGCTCCGCGCTCTGGCGATCTGCCTAAA | omcA -N HR |
| omcA -Ni | AACCCGGTACCGTTCCATCAGGCGTTGAAGCTGCCAGCTGATCTAACTGGTATG | omcA -N HR |
| omcA -Co | AACCCGGGACTTAGTTAGCCTTACAGGTGGG | omcA -C HR |
| omcA -Ci | CATACCAGTTAGATCAGCTGGCAGCTTCAACGCCTGATGGAACGGTACCGGGTT | omcA -C HR |
| cymA-No | AAGAGCTCGGGTTGAAATTCATCCCAAACTC | cymA-N HR |
| cymA -Ni | GCCTTAGCTACTTAAGGTGGTACTTAAAACCATGAAGCCAATGGCTGTGAGAAT | cymA-N HR |
| cymA -Co | AACCCGGGATCTGCTTACTCGCATGACTCAT | cymA-C HR |
| cymA -Ci | ATTCTCACAGCCATTGGCTTCATGGTTTTAAGTACCACCTTAAGTAGCTAAGGC | cymA-C HR |
| Kan^R^: Kanamycin-resistance. Amp^R^: Ampicillin-resistance. Cm^R^ : Chloramphenicol-resistance. | | |
| Gm^R^ : Gentamicin-resistance. *Sma*I (CCCGGG), *Sac*I(GAGCTC), *Xho*I (CTCGAG), restriction sites are underlined. | | |


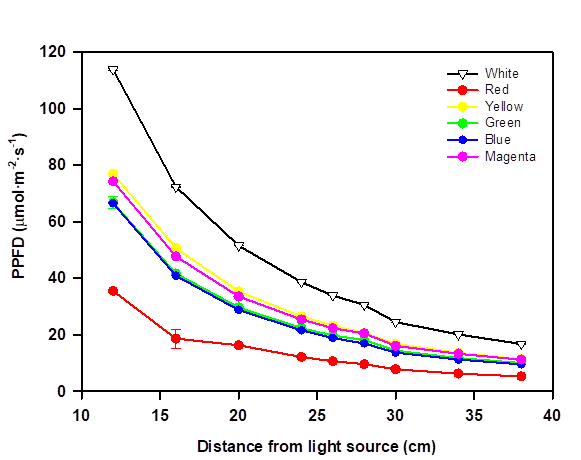

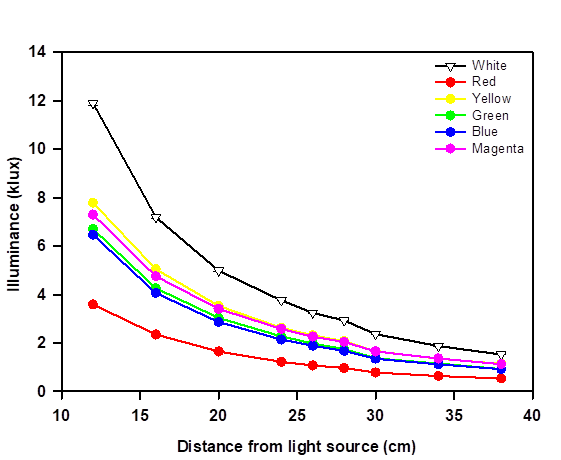


Fig S1 The correlation between distance and light intensity in this study.(A) light intensity in illuminance unit (Lux) (B) light intensity in Photosynthetic photon flux density (μmol/m^2^·s) The number labels stand for the position number and the legend refer the light source.


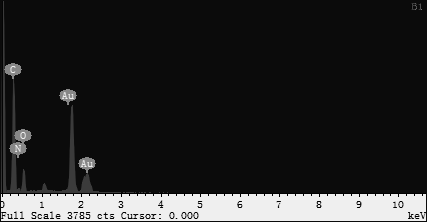

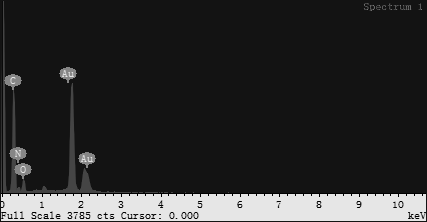


**(B)**

**(A)**

**(D)**


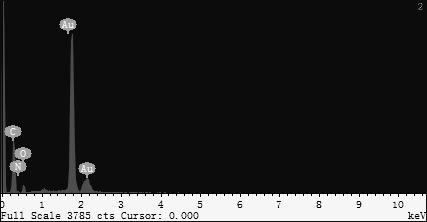

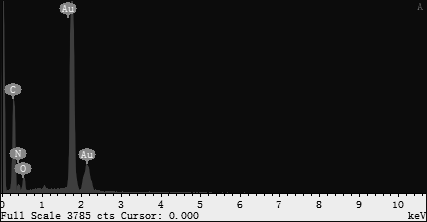


**(D)**

**(C)**

Fig. S2. EDS analysis of *Shewanella oneidensis* MR-1 in condition of different wavelength of light exposure at 25^o^C for 12 h. (A) white light (B) red light (C) blue light (D) green light. The highest peak in the figure represented the existence of gold element.
